# Tenofovir alafenamide induced alopecia in an African American female: A case report to raise awareness on possible racial predisposition

**DOI:** 10.1016/j.jdcr.2025.05.014

**Published:** 2025-06-11

**Authors:** Vivian C. Iloabuchi, Reese L. Imhof, Carilyn Wieland, John D. Zeuli, Silpita Katragadda, Sindhuja Sominidi Damodaran

**Affiliations:** a Mayo Clinic Alix School of Medicine, Scottsdale, Arizona; b Department of Dermatology, Mayo Clinic, Rochester, Minnesota; c Department of Pharmacy, Mayo Clinic, Rochester, Minnesota; d Department of Internal Medicine, Mayo Clinic, Rochester, Minnesota

**Keywords:** antiretroviral, HIV/AIDS, skin of color, telogen effluvium

## Introduction

Alopecia is an infrequent side effect of some anti-retroviral therapy (ART), indinavir and lamivudine generating the most reports through 2014.^1^ Tenofovir alafenamide fumarate (TAF) is a tenofovir (TFV) prodrug approved for HIV in 2015. In comparison to its predecessor, tenofovir disoproxil fumarate (TDF), TAF concentrates TFV in peripheral blood mononuclear cells, and lowers TFV serum exposure, affording less kidney and bone toxicity.^2^ A preferable pharmacodynamic profile supports therapeutic substitution of TAF for TDF. While a true racial predilection of TAF-induced alopecia is unknown, a recent series highlighted alopecia in 6 African American women after switching from TDF to TAF.^3^ We report an additional case of TAF-induced alopecia in an African American woman to highlight the medication side effect and possible racial predisposition.

## Case report

**Fig 1:**
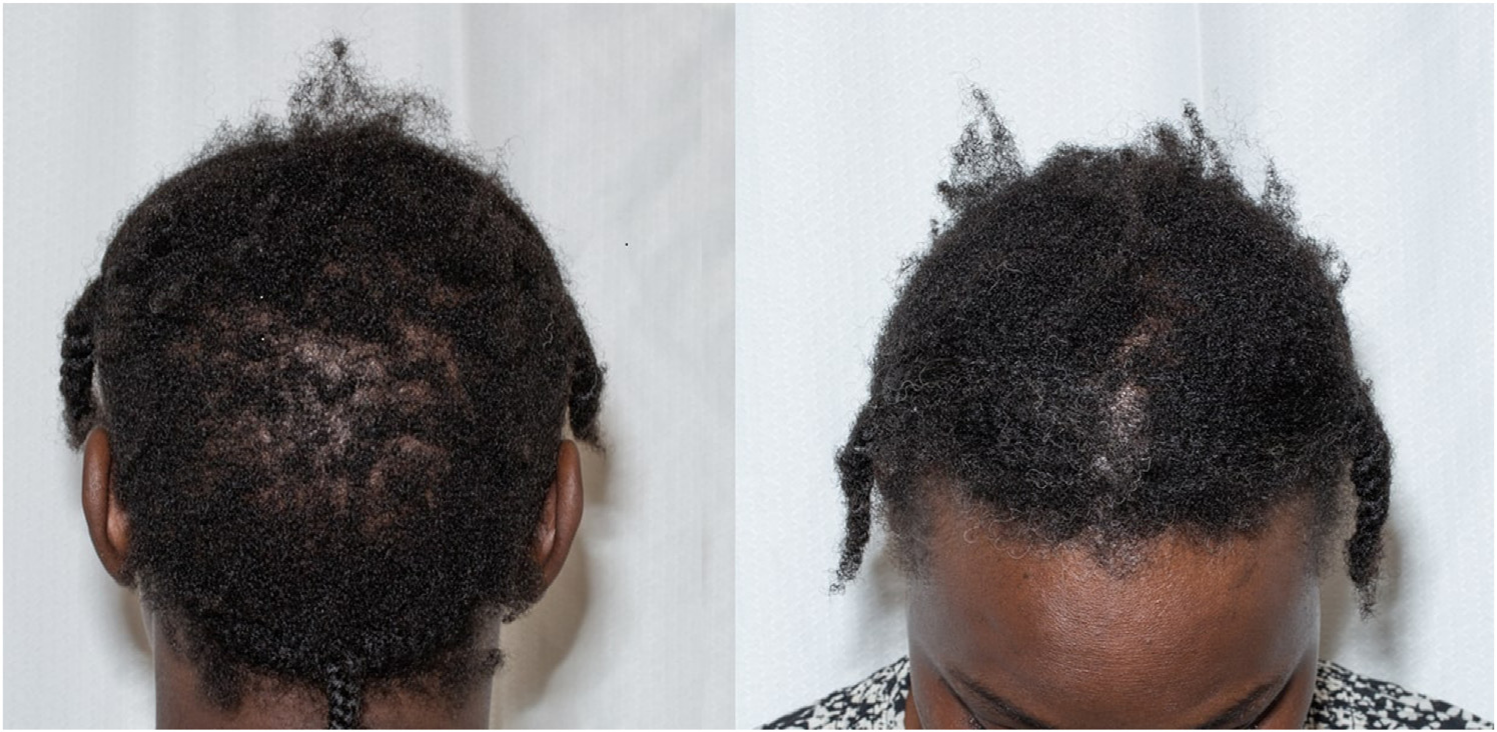
Photographs of the patient’s initial presentation with diffuse thinning throughout the scalp with prominent occipital involvement.

A 44-year-old African American woman with HIV presented for evaluation of hair loss. Previously stable on an HIV regimen of rilpivirine/TDF/emtricitabine, she was switched to bictegravir/TAF/emtricitabine. Two months after the switch, she noticed hair loss (Fig 1). No other side effects were noted, and she maintained viral suppression. Her history was negative for dietary changes or changes in hair styling practices including using styling tools or tight braiding/traction. She was experiencing some life stressors, though these remained continuous before medication change and throughout resolution of her alopecia.

On examination, she had loose braids without evidence for traction. Diffuse thinning throughout the scalp with prominent occipital involvement was noted without discrete alopecic patches (Fig 1). Trichoscopy was negative for signs of scarring or other primary morphology.

**Fig 2:**
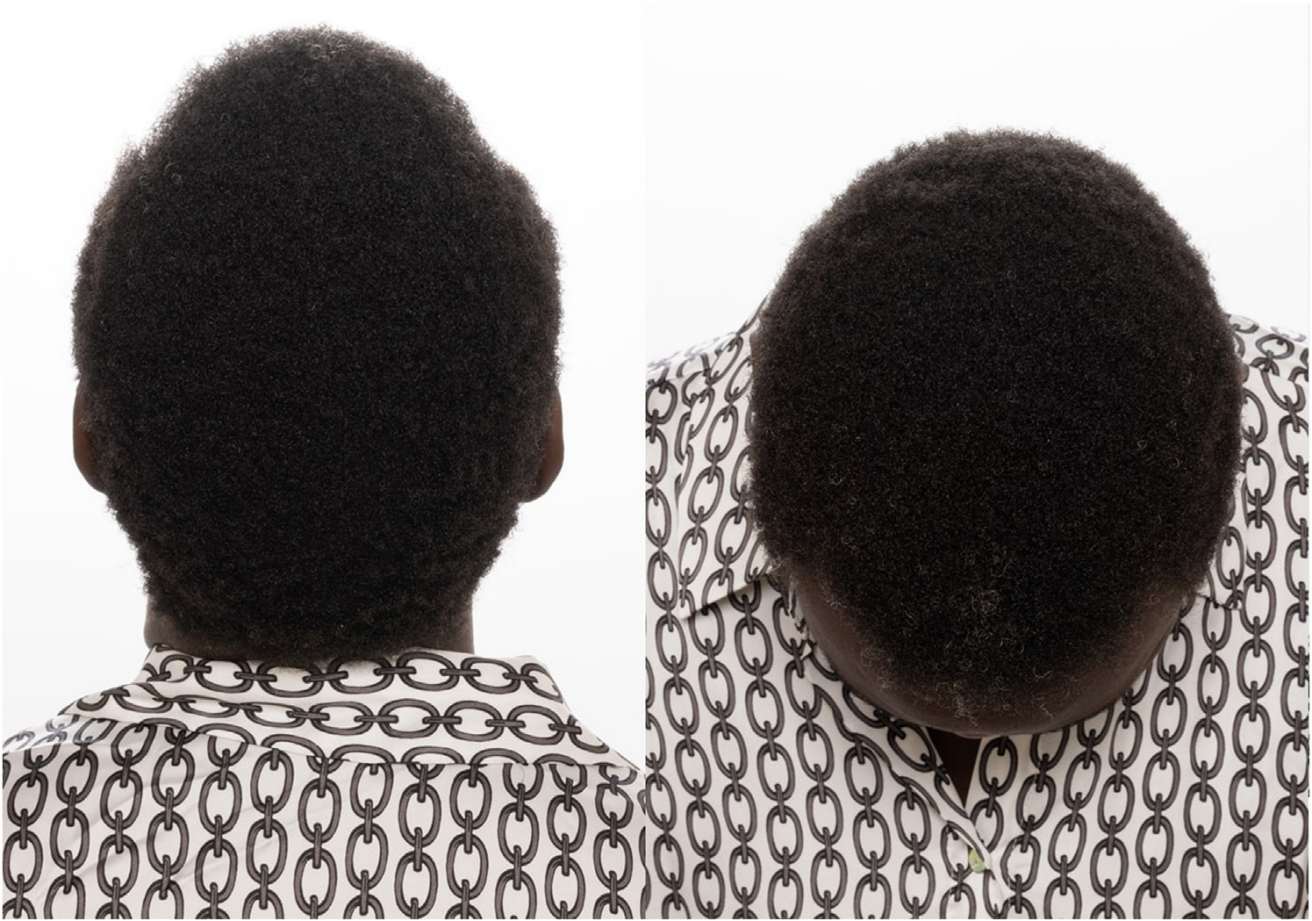
Photographs of the patient 5 months after discontinuing tenofovir alafenamide fumarate (TAF) with complete hair regrowth.

Nutritional work up revealed low ferritin (3 mcg/dL, ref 11-307 mcg/dL), low iron (29 mcg/L, ref 35-145 mcg/L), and borderline low vitamin D (19 ng/ml, ref 20 ng/ml). Further review revealed chronic low ferritin levels for 5 years (3-4 mcg/dL) and low iron (24-29 mcg/L) for 3 years. Nevertheless, iron and vitamin D supplementation was recommended in addition to topical 5% minoxidil solution. Given the timeline of her symptoms, TAF-induced hair loss was suspected, and she was switched to dolutegravir/rilpivirine. She followed up 5 months later with complete hair regrowth (Fig 2).

## Discussion

ART-induced alopecia is an important side effect that may lead to patient distress and potential medication non-adherence. The most common mechanism of ART-induced alopecia is telogen effluvium (TE). Appreciating that neither rilpivirine nor bictegravir are reported to cause alopecia, the timing of hair loss a few months after starting TAF, continued worsening while she remained on TAF, diffuse nonscaring alopecia on examination, and significant improvement after discontinuing TAF are consistent with TAF-induced TE.^4^ Using the Naranjo Adverse Drug Reaction Probability Scale, our patient received a score of +3, which suggests a possible cause-effect relationship between TAF and alopecia. Our patient also had low ferritin, iron, and mild vitamin D deficiency, which are also known causes for alopecia, but the chronicity of these levels makes them unlikely to be causal factors for her acute TE. Additionally, the patient’s life stressors remained present throughout the period immediately prior to and after ART change, continuing through alopecia resolution.

The exact pathogenesis of ART-induced TE is unknown. TAF is a nucleotide reverse transcriptase inhibitor that exerts antiretroviral activity by inhibiting reverse transcriptase. Some nucleotide reverse transcriptase inhibitor adverse effects are caused by inhibition of the human mitochondrial DNA polymerase gamma resulting in mitochondrial dysfunction.^5^ However, why alopecia occurs after switching from TDF to TAF is unclear, as both yield similar TFV concentrations in hair. While adherence to TAF/FTC in the Purpose-1 study was poor, subanalysis showed reduction in HIV infection when taken regularly for HIV pre-exposure prophylaxis.^6^ This could lead to more use of TAF/FTC and additional cases of TAF-induced alopecia.

While hair loss can be multifactorial, published cases to date support TAF may cause alopecia, primarily due to TE, with a racial predilection for African American women. Unfortunately, race or ethnicity is underreported for ART-associated alopecia, making firm conclusions challenging. In Woods et al’s review of 46 ART-associated alopecia case, race or ethnicity was provided for only 3 (a Caucasian woman, a Caucasian man, and a Hispanic man).^1^ In El Zein et al’s case series, 6 African American women were described to have alopecia associated with TAF.^3^ Four of these 6 patients had diffuse nonscarring hair loss within 2-4 months of TAF initiation, consistent with the timeframe and clinical presentation for TE.^3^ Of the 6 patients, one presented with patchy hair loss that was more suspicious for alopecia areata 7 months after TAF initiation.^3^ Our case further supports that TE is the more likely cause of alopecia related to TAF and that African American women may have a higher predisposition for this adverse effect. Future research may help determine the actual mechanism or potential ethnic predisposition of TAF-induced alopecia. Regardless, clinical vigilance is crucial for early diagnosis and management of this distressing, yet reversible, adverse effect.

## Conflicts of interest

None disclosed.
